# Influence of vitamin D supplementation on ovarian reserve as reflected by anti-Müllerian hormone levels: a meta-analysis of randomized controlled trials

**DOI:** 10.3389/fendo.2026.1832704

**Published:** 2026-05-11

**Authors:** Sitian Fang, Yijia Li, Xinyu Fan, Liping Zheng

**Affiliations:** 1 Department of Reproductive Medicine, the 1st affiliated hospital, Jiangxi Medical College, Nanchang University, Nanchang, China; 2 HuanKui Academy, Jiangxi Medical College, Nanchang University, Nanchang, China; 3 School of Humanities & Social Sciences, Gannan Medical University, Ganzhou, China; 4 Queen Mary School, Jiangxi Medical College, Nanchang University, Nanchang, China; 5 Jiangxi Key Laboratory of Reproductive Health, Jiangxi Medical College, Nanchang University, Nanchang, China

**Keywords:** anti-Müllerian hormone, meta-analysis, ovarian reserve, polycystic ovary syndrome, vitamin D

## Abstract

**Background:**

Vitamin D has been implicated in ovarian physiology, yet its effect on ovarian reserve remains controversial. We performed a meta-analysis of randomized controlled trials (RCTs) to evaluate the influence of vitamin D supplementation on ovarian reserve as indicated by serum anti-Müllerian hormone (AMH) levels.

**Methods:**

PubMed, Cochrane Library, Embase, Web of Science, Wanfang, and CNKI were searched for RCTs comparing vitamin D supplementation with placebo or no intervention on AMH in women of reproductive age. The pooled effect was summarized as standardized mean difference (SMD) using a random-effects model by incorporating the influence of potential heterogeneity.

**Results:**

Eleven RCTs with 992 women were included. Overall, vitamin D supplementation did not significantly affect serum AMH levels (SMD: −0.20; 95% CI: −0.48 to 0.08; p = 0.16), with substantial heterogeneity (I² = 77%, p < 0.001). Subgroup analysis showed no significant effect in double-blind trials but a reduction in AMH in open-label trials (p for subgroup difference = 0.02). A significant interaction was observed according to baseline AMH level (p = 0.003), with a reduction in studies where baseline AMH > 6 ng/mL (SMD: −0.55; 95% CI: −0.87 to −0.23). No significant subgroup differences were found by age, baseline 25(OH)D, vitamin D dose, treatment duration, or assay method.

**Conclusions:**

Current randomized evidence suggests that vitamin D supplementation was not likely to alter AMH levels in reproductive-aged women overall. The certainty of evidence was moderate, and findings should be interpreted cautiously given substantial heterogeneity.

**Systematic Review Registration:**

https://www.crd.york.ac.uk/prospero/, identifier CRD420261329210.

## 1 Introduction

Diminished ovarian reserve (DOR) refers to a reduction in the quantity and/or quality of oocytes within the ovary relative to a woman’s age, reflecting impaired reproductive potential (1, 2). Clinically, DOR is characterized by decreased responsiveness to ovarian stimulation, reduced antral follicle count (AFC), elevated follicle-stimulating hormone (FSH), and particularly reduced serum anti-Müllerian hormone (AMH) levels (3, 4). The prevalence of DOR varies depending on diagnostic criteria and population, but it is estimated to affect approximately 10–25% of women seeking infertility treatment, with increasing incidence in women of advanced reproductive age (5). DOR is associated with lower spontaneous conception rates, poor response to assisted reproductive technologies, higher cycle cancellation rates, and reduced live birth rates, thereby imposing substantial emotional and economic burdens (6–9). Among available biomarkers, AMH is widely used as a surrogate indicator of ovarian reserve. It is secreted by granulosa cells of pre-antral and small antral follicles and exhibits minimal intra-cycle variability. However, AMH primarily reflects the population of growing follicles—often referred to as functional ovarian reserve—rather than directly measuring the primordial follicle pool (10, 11). However, AMH interpretation may differ between clinical contexts. In women with polycystic ovary syndrome (PCOS), AMH levels are typically elevated due to increased small follicle numbers and granulosa cell dysfunction; whereas in non-PCOS women, lower AMH levels more directly reflect follicular depletion (12, 13). These distinct pathophysiological backgrounds suggest that factors influencing AMH may exert differential effects in PCOS and non-PCOS populations.

Vitamin D, a secosteroid hormone primarily synthesized in the skin and subsequently hydroxylated to its active form, exerts pleiotropic effects beyond calcium homeostasis (14). Vitamin D receptors and vitamin D–metabolizing enzymes are expressed in ovarian tissue, including granulosa cells, suggesting a potential role in folliculogenesis and steroidogenesis (15). Experimental evidence indicates that vitamin D may regulate AMH gene expression, modulate FSH sensitivity, and influence ovarian steroid hormone production (16, 17). Observational studies have reported associations between low serum 25-hydroxyvitamin D [25(OH)D] levels and impaired ovarian reserve, including lower AMH levels or increased risk of DOR, although findings remain inconsistent (17–19). Furthermore, interventional trials evaluating the effect of vitamin D supplementation on AMH have yielded heterogeneous results, with some studies reporting increases (20, 21), others decreases (22–25), and many showing no significant change (26–30). These discrepancies may reflect differences in baseline ovarian reserve status, vitamin D deficiency severity, dosing regimens, and study design, particularly between PCOS and non-PCOS populations. Therefore, we conducted a systematic review and meta-analysis of randomized controlled trials to comprehensively evaluate the effect of vitamin D supplementation on ovarian reserve as indicated by serum AMH levels and to explore potential sources of heterogeneity across subgroups.

## 2 Methods

This systematic review and meta-analysis followed established methodological guidance from the PRISMA (Preferred Reporting Items for Systematic Reviews and Meta-Analyses) reporting framework (31) and the Cochrane Handbook for Systematic Reviews of Interventions (32). The review protocol was registered prospectively with the PROSPERO international prospective register of systematic reviews (CRD420261329210).

### 2.1 Study inclusion and exclusion criteria

Eligible studies were selected based on predefined criteria structured according to the PICOS framework. Study selection was performed independently by two reviewers, who screened titles, abstracts, and full texts according to the predefined eligibility criteria. Discrepancies were resolved through discussion with the corresponding author.

P (Population): Women of reproductive age, including healthy women or those with reproductive conditions (e.g. infertility, diminished ovarian reserve, or polycystic ovary syndrome [PCOS]), in whom serum AMH levels were assessed. Studies must involve non-pregnant, premenopausal women.

I (Intervention): Vitamin D supplementation administered as cholecalciferol (vitamin D3), ergocalciferol (vitamin D2), or active vitamin D analogues (e.g., calcitriol, alfacalcidol), at any dose, frequency, or duration.

C (Comparator): Placebo, no treatment, or standard care without vitamin D supplementation. Co-interventions are acceptable if applied equally across study groups (balanced).

O (Outcome): Serum AMH levels reported as post-intervention values and/or change from baseline, with sufficient data to calculate effect estimates.

S (Study Design): RCTs with parallel group published as full-text articles in peer-reviewed journals. Only peer-reviewed published studies were included to ensure data reliability and allow formal assessment of study quality and risk of bias.

Studies were excluded if they were observational studies (cohort, case-control, cross-sectional), non-randomized trials, quasi-experimental studies, reviews, meta-analyses, editorials, animal or *in vitro* studies; if they did not report extractable AMH data; if vitamin D was combined with other active interventions in an unbalanced manner between groups; if participants were pregnant, postmenopausal, or had undergone oophorectomy; or if duplicate or overlapping populations were identified (in which case the study with the largest sample size was included).

### 2.2 Database search

PubMed, Cochrane Library, Embase, Web of Science, Wanfang, and China National Knowledge Infrastructure (CNKI) were systematically searched using the combined terms including (1): “vitamin D” OR “vitamin D2” OR “vitamin D3” OR “cholecalciferol” OR “ergocalciferol” OR “alphacalcidol” OR “alfacalcidol” OR “calcitriol” OR “paricalcitol” OR “doxerocalciferol” (2); “anti-Müllerian hormone” OR “anti Mullerian hormone” OR “AMH”; and (3) “randomized controlled trial” OR “randomised controlled trial” OR “randomized” OR “randomised” OR “RCT” OR “randomly” OR “placebo” OR “control” OR “allocated” OR “allocation”. We included peer-reviewed full-text studies published in English or Chinese, as these were the languages accessible to the review team for accurate data extraction and quality assessment. In addition, the bibliographies of relevant reviews and eligible articles were manually screened to identify any additional records. The final search update was performed on December 26, 2025, and the detailed database-specific search strategies are provided in **Supplementary File 1**.

### 2.3 Study quality evaluation

The quality of the included RCTs was assessed using the Cochrane Risk of Bias tool (RoB 2), which evaluates potential bias across five key domains: the randomization procedure, deviations from intended interventions, completeness of outcome data, outcome measurement, and selective reporting of results (32). Each domain was judged as presenting low risk, some concerns, or high risk of bias, and these assessments were combined to determine an overall risk-of-bias rating for each study.

### 2.4 Data extraction

Data extraction was independently performed by two reviewers using a standardized form. Any disagreements were resolved through discussion with the corresponding author. Extracted data included general study information (first author, publication year, and country), study design (double-blind, single-blind, or open-label), participant characteristics (diagnosis, numbers of women included, mean age, baseline mean vitamin D level as indicated by 25-hydroxyvitamin D [25(OH)D], baseline mean AMH level, details of vitamin D supplementation, average daily dose of vitamin D, details of control, details of concurrent treatment, duration of intervention, and methods for measuring the serum level of AMH. For studies reporting variability as standard error (SEM), SDs were calculated using the formula SD = SEM × √n. Where necessary, AMH values were converted to a common unit (ng/mL) using standard conversion factors (1 pM = 0.14 ng/mL) to ensure consistency across studies.

### 2.5 Statistical analysis

The influence of vitamin D supplementation on AMH level in women as compared to controls was summarized as standardized mean difference (SMD) and 95% confidence intervals (CIs), because the units and methods for measuring AMH varied among the included studies (32). Statistical heterogeneity across studies was examined using Cochran’s Q test and further quantified with the I² statistic, with values below 25%, between 25% and 75%, and above 75% reflecting low, moderate, and substantial heterogeneity, respectively (33). Pooled estimates were generated using a random-effects model to account for anticipated clinical and methodological diversity among trials (32). The stability of the findings was evaluated through leave-one-out analyses (32). Prespecified subgroup analyses were performed based on participant and trial characteristics, including study design (open-label vs. double-blind), diagnosis (PCOS vs. non-PCOS), mean ages of the women, baseline 25(OH)D level, baseline mean AMH level, daily dose of vitamin D, treatment duration, and methods for measuring the serum level of AMH. For continuous moderators, median values were applied as cut points to achieve balanced subgroup distributions. A symmetrical funnel plot was considered to indicate a low likelihood of small-study effects, whereas asymmetry may suggest potential publication bias or other sources of heterogeneity. Given the relatively small number of included studies, the results of Egger’s test were interpreted with caution. Potential publication bias was assessed by visual inspection of funnel plot symmetry and further evaluated using Egger’s regression test (34). A symmetrical funnel plot was considered to indicate a low likelihood of small-study effects, whereas asymmetry may suggest potential publication bias or other sources of heterogeneity. Given the relatively small number of included studies, the results of Egger’s test were interpreted with caution. Statistical significance was defined as a two-sided *p* value < 0.05. All analyses were carried out using RevMan (version 5.3, Cochrane Collaboration, Oxford, UK) and Stata (version 17.0, StataCorp, College Station, TX, USA) software.

### 2.6 Certainty of evidence

The overall certainty of evidence was independently evaluated by two reviewers using the GRADE (Grading of Recommendations, Assessment, Development and Evaluation) framework, which rates confidence in the findings across domains including risk of bias, inconsistency, indirectness, imprecision, and potential publication bias (35). The certainty of evidence was categorized as high, moderate, low, or very low, and any differences in judgment were resolved through discussion and agreement.

## 3 Results

### 3.1 Literature search

**Figure 1:**
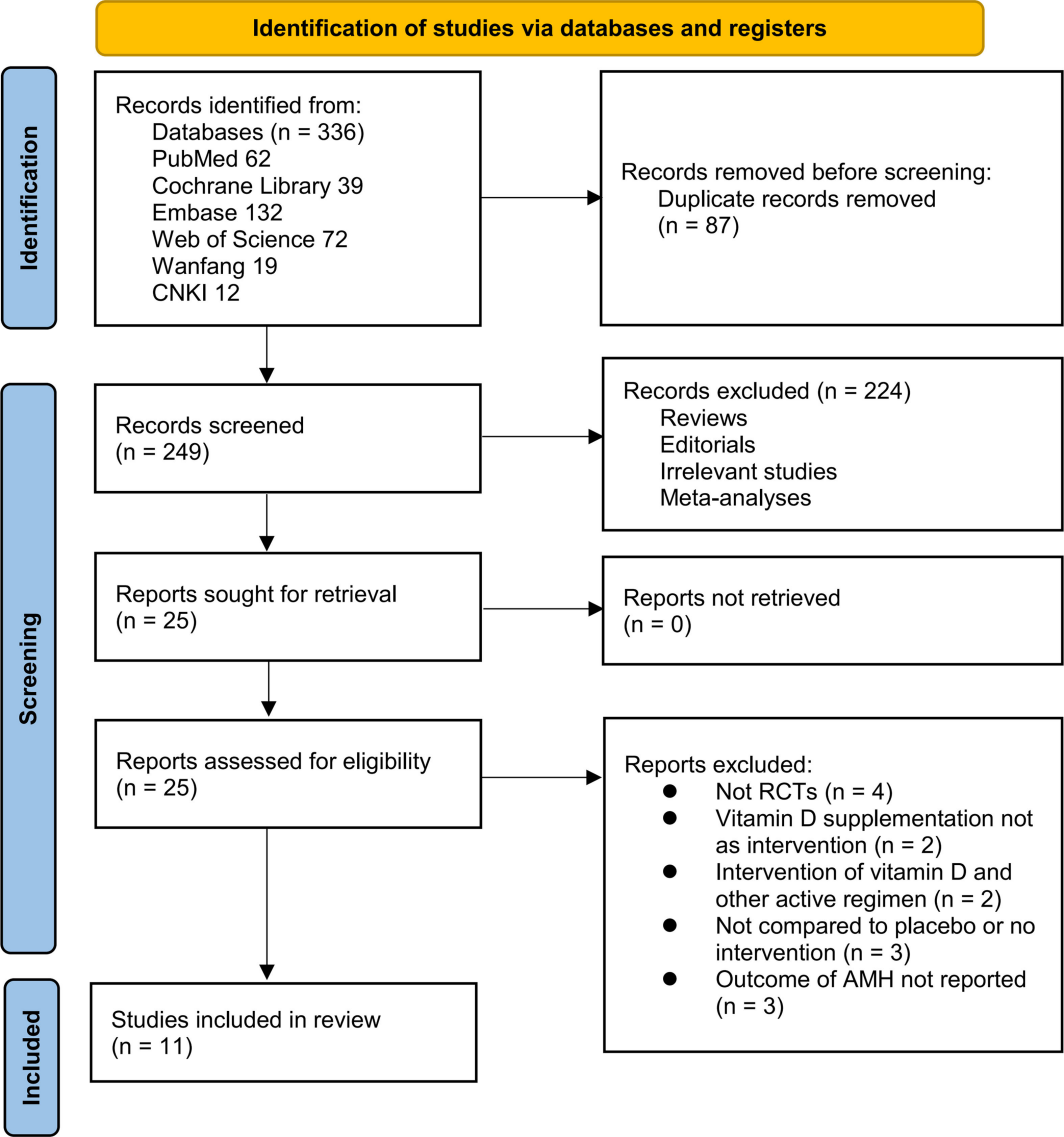
Flowchart for the literature search and study inclusion.

**Figure 1** depicts the flowchart that outlines the process of database searching and study identification, ultimately leading to the selection of studies for inclusion. Initially, a total of 336 articles were obtained through the database search, which was subsequently reduced to 249 after eliminating duplicate records. Subsequently, 224 articles were excluded based on an evaluation of their titles and abstracts, primarily due to their lack of relevance to the objective of the present meta-analysis. The remaining 25 full-text articles were assessed, and 14 were excluded for reasons detailed in **Figure 1**. Ultimately, 11 RCTs (20–30) were deemed suitable for quantitative analysis.

### 3.2 Characteristics of the included studies

The main characteristics of the included randomized controlled trials (RCTs) are summarized in **Table 1**. This meta-analysis incorporated 11 RCTs published between 2015 and 2025, conducted across Asia (China, Iran, Bangladesh), Europe (Austria), and Oceania (New Zealand). Because two trials (21, 27) included separate PCOS and non-PCOS cohorts, these were treated as independent comparisons, resulting in 13 analyzable datasets. Overall, the included studies enrolled women with diverse clinical backgrounds, predominantly PCOS, but also infertile women without PCOS, women with DOR, healthy premenopausal women, and breast cancer patients undergoing chemotherapy. Sample sizes ranged from 17 to 150 participants per study, with a total number of 992 women included. The mean age of participants varied approximately from 21.7 to 35.8 years, reflecting predominantly reproductive-age populations. Baseline mean serum 25(OH)D levels, when reported, generally indicated insufficiency or deficiency (approximately 10.75–22.19 ng/mL). Baseline AMH levels varied widely depending on the population, with markedly elevated values in PCOS cohorts (often >5 ng/mL) and low levels in DOR patients (mean AMH approximately 0.44 ng/mL). All interventions involved oral vitamin D3 (cholecalciferol), with considerable variation in dosing regimens. Daily-equivalent doses ranged from 200 IU/day to 12,000 IU/day, and some studies used intermittent high-dose strategies (e.g., 50,000 IU every two weeks (22, 23) or 40,000 IU once weekly (28)). Treatment durations ranged from 1 week (single-dose study) to 24 weeks. Control groups received either placebo (in double-blind trials) (20–22, 27, 30) or no intervention (in open-label studies) (23–26, 28, 29). In four studies (24–26, 30), concurrent treatments were administered, including Diane-35 (ethinylestradiol–cyproterone acetate), metformin, DHEA, or inositol, although these were typically balanced between groups. AMH was measured using enzyme-linked immunosorbent assay (ELISA) (20, 22, 23, 27, 29, 30) or chemiluminescence-based methods (CLIA or electrochemiluminescence platforms) (21, 24, 25, 28), reflecting some methodological heterogeneity in laboratory assessment. The method for measuring serum AMH was not reported in detail in another study (26).

**Table 1:** Characteristics of the included studies.

Study	Country	Design	Diagnosis	No. of women included	Mean age (years)	Baseline mean 25(OH)D (ng/mL)	Baseline mean AMH (ng/mL)	Details of VitD supplementation	Daily dose of VitD3 (IU)	Details of control	Concurrent treatment	Treatment duration (weeks)	Methods for measuring AMH
Zhang 2015 (26)	China	R, OL	PCOS	60	26.5	21.35	9.75	Oral VitD3, 12,000 IU daily	12,000	No intervention	Diane-35	12	NR
Dennis 2017 (20)	New Zealand	R, DB, PC	Healthy women without endocrine or reproductive diseases, including PCOS	46	21.7	21.15	5.01	Single oral dose of VitD3, 50,000 IU	7,143	Placebo	None	1	ELISA
Dastorani 2018 (22)	Iran	R, DB, PC	Infertile women with PCOS	40	30.0	10.75	8.20	Oral dose of VitD3, 50,000 IU/2 weeks	3,571	Placebo	None	8	ELISA
Hosseini 2019 (23)	Iran	R, OL	PCOS	17	28.9	NR	12.23	Oral dose of VitD3, 50,000 IU/2 weeks	3,571	No intervention	None	8	ELISA
Lerchbaum 2021 PCOS (27)	Austria	R, DB, PC	PCOS	120	26.0	20.19	7.67	Oral VitD3 20,000 IU once weekly	2,857	Placebo	None	24	ELISA
Lerchbaum 2021 non-PCOS (27)	Austria	R, DB, PC	Healthy premenopausal women without PCOS	127	35.8	22.19	1.97	Oral VitD3 20,000 IU once weekly	2,857	Placebo	None	24	ELISA
Wei 2021 (24)	China	R, OL	Infertile women with PCOS	72	28.2	18.31	6.92	Oral VitD3, 200 IU daily	200	No intervention	Metformin and Diane-35	12	CLIA
Zhou 2022 (25)	China	R, OL	PCOS	110	27.2	NR	13.99	Oral VitD3, 400 IU daily	400	No intervention	Diane-35	12	CLIA
Halder 2022 (28)	Bangladesh	R, OL	Infertile women with DOR, defined as serum AMH < 1 ng/mL	44	33.8	NR	0.44	Oral VitD3, 40,000 IU once weekly	5,714	No intervention	DHEA	8	CLIA
Li 2022 PCOS (21)	China	R, DB, PC	Infertile women with PCOS and vitamin D deficiency (25(OH)D < 20 ng/mL)	150	27.6	15.07	5.42	Oral vitamin D3 drops, 2,000 IU daily	2,000	Placebo	None	12	CLIA
Li 2022 non-PCOS (21)	China	R, DB, PC	Infertile women without PCOS, with vitamin D deficiency (25(OH)D < 20 ng/mL)	120	28.0	15.50	5.51	Oral vitamin D3 drops, 2,000 IU daily	2,000	Placebo	None	12	CLIA
Dastmardi 2024 (29)	Iran	R, OL	Breast cancer patients (aged 18–45 years) undergoing adjuvant or neoadjuvant chemotherapy	33	34.9	NR	2.80	Oral vitamin D3, 1,000 IU daily	1,000	No intervention	None	24	ELISA
Banikazemi 2025 (30)	Iran	R, DB, PC	PCOS	53	33.7	NR	5.28	Oral VitD3, 4,000 IU daily	4,000	Placebo	Inositol	8	ELISA

R, randomized; OL, open-label; DB, double-blind; PC, placebo-controlled; PCOS, polycystic ovary syndrome; DOR, diminished ovarian reserve; AMH, anti-Müllerian hormone; 25(OH)D, 25-hydroxyvitamin D; VitD, vitamin D; VitD3, vitamin D3 (cholecalciferol); IU, international units; NR, not reported; DHEA, dehydroepiandrosterone; CLIA, chemiluminescence immunoassay; ELISA, enzyme-linked immunosorbent assay.

### 3.3 Study quality evaluation

Risk of bias assessment using the Cochrane Risk of Bias 2.0 tool is summarized in **Table 2**. Overall, the methodological quality of the included RCTs was acceptable. The randomization process was judged as low risk in the majority of studies, with most trials employing computer-generated randomization, block randomization, or random number tables, and reporting balanced baseline characteristics. Allocation concealment was explicitly described in all the double-blind, placebo-controlled trials (20–22, 27, 30). Regarding deviations from intended interventions, open-label designs were common, particularly in Chinese and Iranian trials, resulting in “some concerns” for this domain in several studies. However, because the intervention consisted of oral vitamin D supplementation and AMH is an objective laboratory outcome, the potential impact of performance bias was likely limited. Double-blind, placebo-controlled trials (20–22, 27, 30) were consistently rated as low risk in this domain. Most studies were judged as low risk for missing outcome data, as follow-up was complete or dropout rates were low and balanced between groups. However, some trials (e.g., Lerchbaum 2021 PCOS cohort (27) and Halder 2022 (28)) exhibited relatively high attrition rates or used per-protocol analyses, leading to some concerns regarding potential attrition bias. Measurement of the outcome was generally robust. AMH was assessed using validated ELISA or chemiluminescence-based assays with reported coefficients of variation, and laboratory measurements are inherently objective. All the double-blind trials (20–22, 27, 30) ensured assessor blinding, resulting in low risk ratings for outcome measurement across nearly all studies. Selective reporting bias was considered unlikely in all trials, as reported outcomes corresponded to prespecified endpoints described in the methods sections. In summary, all the included RCTs were judged as low risk of bias (21–23, 27, 30) or having some concerns (23–29), primarily driven by open-label design or moderate attrition. High risk of bias was not identified. Overall, the evidence base consists of randomized trials with objective biochemical outcome assessment, while acknowledging unavoidable limitations related to lack of blinding in several studies and heterogeneity in dosing regimens and clinical populations.

**Table 2:** Study quality evaluation via the RoB 2.0 Tool.

Study	Randomization process	Deviations from intended interventions	Missing outcome data	Measurement of the outcome	Selection of the reported results	Overall
Zhang 2015 (26)	Low risk (Randomization using a random number table, no baseline imbalances were reported)	Some concerns (The study was open-label as the control group did not receive a placebo. Participants and personnel were likely aware of the intervention assignment. While this could lead to deviations, the intervention was a simple supplement, and the co-intervention (Diane-35) was identical in both groups)	Low risk (The study reports results for 30 patients in each group at the end of the trial, suggesting complete follow-up for the primary outcomes. No missing data is mentioned)	Low risk (The outcome (AMH) was measured using a serum sample, which is objective. The study does not state if the assessors were blinded, but as a laboratory measurement, it is unlikely to be influenced by knowledge of the intervention)	Low risk (The study reports on multiple outcomes (hormones, glucose, insulin, ultrasound findings) as stated in the methods)	Some concerns
Dennis 2017 (20)	Low risk (The study used a permuted block randomization method with a block size of ten. Study capsules were coded by a third party. No baseline imbalances were observed between groups for any parameters)	Low risk (The study was double-blind (participants and investigators remained blinded until after statistical analyses). A research nurse observed the consumption of the capsule, ensuring adherence. There is no mention of deviations from the intended intervention)	Low risk (Outcome data appears to be complete for the analyzed participants. Three women were excluded *post-hoc* due to LH surge (pre-specified criteria), which is appropriate and unlikely to introduce bias. AMH data was reported for all 24 (Vit D) and 22 (placebo) participants at all time point)	Low risk (The outcome (AMH) was measured using a validated ELISA method. All samples were analyzed as a single batch to avoid inter-assay variation (intra-assay CV <6%). The outcome assessors were blinded to group allocation)	Low risk (The paper reports on the pre-specified outcomes (change in AMH and 25(OH)D over time). The results are presented in figures and text consistent with the stated aims. The analysis plan (using log ratios to normalize data) was clearly described and appropriate)	Low risk
Dastorani 2018 (22)	Low risk (The study used a random number table for randomization. Randomization and allocation were concealed from investigators and participants until after analyses. No significant baseline differences were observed between groups for age, weight, BMI, or primary outcomes)	Low risk (The study was double-blind (participants and investigators were blinded). Compliance was high (90-100%) and was assessed by asking patients to return medication containers and by measuring serum 25(OH)D levels. No side effects were reported. Analysis was by ITT)	Low risk (Although 3 participants dropped out from each group (6 total), the authors performed an ITT analysis including all 40 randomized participants. This appropriately handles missing data and minimizes bias)	Low risk (The outcome (AMH) was measured using a validated ELISA method with low CVs (<6%). The outcome assessors were blinded to group allocation)	Low risk (The paper reports on the pre-specified outcomes listed in the methods (AMH, glycemic control, lipid profiles, gene expression). Results are presented clearly in tables and figures consistent with the stated aims)	Low risk
Hosseini 2019 (23)	Some concerns (The study states participants were “randomly divided into six groups,” but the method of randomization (e.g., random number table, computer generation) is not specified. Baseline characteristics appear similar, but the lack of detail raises some concerns)	Some concerns (The study was open-label (no blinding mentioned). Participants and personnel were aware of the intervention assignment. This could influence other behaviors. However, the vitamin D-only and control groups are being compared, and no co-interventions are mentioned)	Low risk (The study reports 60 enrolled and 54 completed (6 dropouts across all groups). For the vitamin D-only group, 1 dropout occurred (from 10 to 9). For the control group, 2 dropouts occurred (from 10 to 8). The numbers are small and relatively balanced)	Low risk (The AMH was measured using a validated ELISA method. The open-label design is unlikely to influence this laboratory measurement)	Low risk (The paper reports on AMH and BMI outcomes as stated in the methods. Results are presented in tables consistent with the aims)	Some concerns
Lerchbaum 2021 PCOS (27)	Low risk (The study used a computer-generated randomization list with a 2:1 ratio. Allocation was concealed. Baseline characteristics were generally similar between groups)	Low risk (The study was double-blind (participants and investigators masked). Compliance was verified by asking participants to return empty medication bottles. Analysis was by ITT)	Some concerns (Of 180 randomized, 123 completed both visits (68.3% completion rate). For AMH, data was available for 80/119 (67%) in VD group and 40/61 (66%) in PBO group. The dropout rate is relatively high, though balanced between groups)	Low risk (The outcome (AMH) was measured using validated ELISA methods with CV <10%. Assay change during the study was handled by confirming good correlation (r=0.95). Outcome assessors were masked)	Low risk (The paper reports on pre-specified outcomes. AMH results are clearly presented in **Table 2** with treatment effects and confidence intervals)	Some concerns
Lerchbaum 2021 non-PCOS (27)	Low risk (The study used a computer-generated randomization list with a 2:1 ratio. Allocation was concealed. Baseline characteristics were generally similar between groups)	Low risk (The study was double-blind (participants and investigators masked). Compliance was verified by asking participants to return empty medication bottles. Analysis was by ITT)	Low risk (Of 150 randomized, 127 completed both visits (84.7% completion rate). This is a reasonable completion rate with balanced dropout)	Low risk (The outcome (AMH) was measured using validated ELISA methods with CV <10%. Assay change during the study was handled by confirming good correlation (r=0.95). Outcome assessors were masked)	Low risk (The paper reports on pre-specified outcomes. AMH results are clearly presented in **Table 2** with treatment effects and confidence intervals)	Low risk
Wei 2021 (24)	Low risk (The study explicitly states it used a random number table method to assign patients. Baseline characteristics (age, infertility duration, hormone levels) were similar between groups with no statistically significant differences)	Some concerns (The study was open-label (no placebo used). Participants and personnel were aware of the intervention assignment. However, the intervention was a simple supplement, and the co-interventions were identical in both groups. There is no mention of deviations)	Low risk (All 72 enrolled patients (36 in each group) completed the study and were included in the analysis. No dropouts or missing data are reported)	Low risk (The outcome (AMH) was measured using a standardized laboratory method (electrochemiluminescence immunoassay). The open-label design is unlikely to influence this objective laboratory measurement)	Low risk (The paper reports on all outcomes mentioned in the methods (hormonal profiles, insulin resistance rate, pregnancy outcomes). The AMH results are clearly presented in **Table 1**)	Some concerns
Zhou 2022 (25)	Low risk (The study explicitly states it used a random number table method to assign patients. Baseline characteristics (age, disease duration, BMI) were similar between groups with no statistically significant differences)	Some concerns (The study was open-label (no placebo used). Participants and personnel were aware of the intervention assignment. However, the intervention was a simple supplement, and the co-intervention (Diane-35) was identical in both groups. There is no mention of deviations)	Low risk (All 110 enrolled patients (55 in each group) completed the study and were included in the analysis. No dropouts or missing data are reported)	Low risk (The outcome (AMH) was measured using a standardized laboratory method (immunofluorescence). The open-label design is unlikely to influence this objective laboratory measurement)	Low risk (The paper reports on all outcomes mentioned in the methods (lipid metabolism, sex hormones, oxidative stress). The AMH results are clearly presented in **Table 2**)	Some concerns
Halder 2022 (28)	Low risk (The study used computer-generated random numbers with permuted block randomization stratified for age. Allocation concealment was done using sequentially numbered sealed opaque envelopes. Baseline characteristics were similar between groups)	Some concerns (The study was open-label (no placebo used). Participants and personnel were aware of the intervention assignment. However, the intervention was a simple supplement, and the co-intervention DHEA was identical in both groups. There is no mention of deviations)	Some concerns (Of 67 randomized, 44 completed (65.7% completion rate). Dropout rate was 33.3% (12/36) in the vitamin D group and 35.5% (11/31) in the control group. Reasons for dropout are reported (lost to follow-up, pregnancy, non-compliance), but the high and unbalanced (in reasons) dropout rate introduces risk of bias. Analysis was per-protocol, not ITT)	Low risk (The outcome (AMH) was measured using a validated automated chemiluminescence immunoassay. Although outcome assessors were not blinded, this laboratory measurement is unlikely to be influenced by lack of blinding)	Low risk (The paper reports on the pre-specified outcomes (change in AMH, AFC, FSH). Results are presented in tables consistent with the aims)	Some concerns
Li 2022 PCOS (21)	Low risk (The study used a random number table method to assign patients. Baseline characteristics (age, BMI, baseline 25(OH)D) were similar between groups with no statistically significant differences)	Low risk (A placebo was used, and it was described as “indistinguishable” in appearance from the vitamin D drops. This suggests blinding of participants. The intervention was a simple supplement with no reported deviations)	Low risk (The study reports results for 75 patients in each group at the end of the trial, suggesting complete follow-up for the primary outcomes. No missing data is mentioned)	Low risk (The outcome (AMH) was measured using a standardized automated chemiluminescence immunoassay. The use of a placebo suggests outcome assessors were likely blinded)	Low risk (The paper reports on the outcomes mentioned in the methods (hormonal profiles, menstrual recovery, ovulation, pregnancy). The AMH results are presented in **Table 3**)	Low risk
Li 2022 non-PCOS (21)	Low risk (The study used a random number table method to assign patients. Baseline characteristics (age, BMI, baseline 25(OH)D) were similar between groups with no statistically significant differences)	Low risk (A placebo was used, and it was described as “indistinguishable” in appearance from the vitamin D drops. This suggests blinding of participants. The intervention was a simple supplement with no reported deviations)	Low risk (The study reports results for 75 patients in each group at the end of the trial, suggesting complete follow-up for the primary outcomes. No missing data is mentioned)	Low risk (The outcome (AMH) was measured using a standardized automated chemiluminescence immunoassay. The use of a placebo suggests outcome assessors were likely blinded)	Low risk (The paper reports on the outcomes mentioned in the methods (hormonal profiles, menstrual recovery, ovulation, pregnancy). The AMH results are presented in **Table 3**)	Low risk
Dastmardi 2024 (29)	Low risk (The study used a web-based computer program with permuted block randomization method. Baseline characteristics (age, BMI, parity, tumor characteristics) were similar between groups)	Some concerns (The study was open-label (no blinding mentioned). Participants and personnel were aware of the intervention assignment. This could influence other behaviors. However, the intervention was a simple supplement, and the co-intervention (chemotherapy) was standardized)	Some concerns (The target sample size was 40, but only 33 were enrolled due to COVID-19. The study stopped early, which may introduce bias. However, all enrolled patients appear to have completed the study and were analyzed)	Low risk (The outcome (AMH) was measured using a validated ELISA method. The open-label design is unlikely to influence this laboratory measurement)	Low risk (The paper reports on the pre-specified outcomes (AMH at baseline, after chemotherapy, and 6 months post-chemotherapy). Results are clearly presented in tables)	Some concerns
Banikazemi 2025 (30)	Low risk (The study used computer-generated random numbers. Randomization was stratified by BMI and age. Allocation was concealed from participants and researchers until final analyses. Baseline characteristics (age, BMI, weight) were similar between groups)	Low risk (The study was double-blind (participants, researchers, and statistical analyzer were blinded). Placebo capsules were indistinguishable from supplements. Adherence was likely monitored (though not detailed), and analysis was by ITT)	Low risk (Of 120 randomized, 103 completed (85.8% completion rate). Reasons for dropout were personal reasons and not meeting criteria for follicular puncture. Dropouts were relatively balanced across groups)	Low risk (The outcome (AMH) was measured using a validated ELISA method with low CV (<7%). Outcome assessors were blinded)	Low risk (The paper reports on all pre-specified outcomes (AMH, glycemic control, inflammatory markers). AMH results are clearly presented in **Table 3** and 4)	Low risk

RoB 2.0, Risk of Bias 2.0 tool; R, randomized; OL, open-label; DB, double-blind; PC, placebo-controlled; PCOS, polycystic ovary syndrome; DOR, diminished ovarian reserve.

AMH, anti-Müllerian hormone; 25(OH)D, 25-hydroxyvitamin D; BMI, body mass index; LH, luteinizing hormone; FSH, follicle-stimulating hormone; AFC, antral follicle count; DHEA, dehydroepiandrosterone; ITT, intention-to-treat; PBO, placebo; CV, coefficient of variation; IU, international units; NR, not reported.

### 3.4 Meta-analysis results

**Figure 2:**
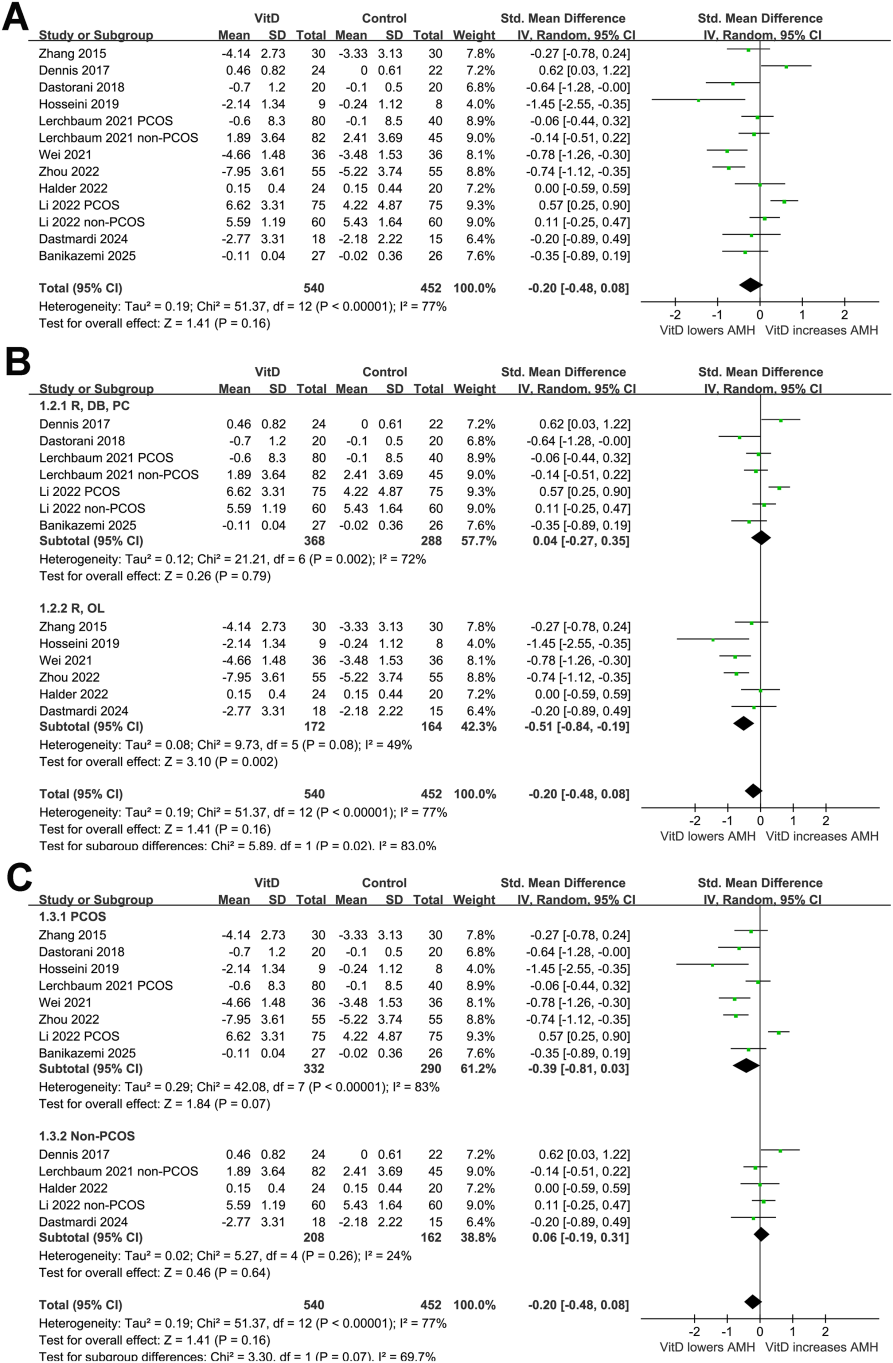
Forest plots for the meta-analysis evaluating the influence of vitamin D supplementation on serum AMH level in women of reproductive age; **(A)** overall meta-analysis; **(B)** subgroup analysis according to study design; and **(C)** subgroup analysis according to the diagnosis of PCOS.

Overall, this meta-analysis included 13 comparisons from 11 RCTs (20–30) and involved 540 women receiving vitamin D supplementation and 452 women receiving placebo or no additional intervention. The pooled results with a random-effects model showed that overall, vitamin D supplementation did not significantly affect the serum level of AMH in women (SMD: -0.20, 95% CI: -0.48 to 0.08, *p* = 0.16; **Figure 2A**) with significant heterogeneity (*p* for Cochrane Q test < 0.001, I^2^ = 77%). The sensitivity analysis by excluding one dataset at a time showed consistent results (SMD: -0.14 to -0.27, *p* all > 0.05).

**Figure 3:**
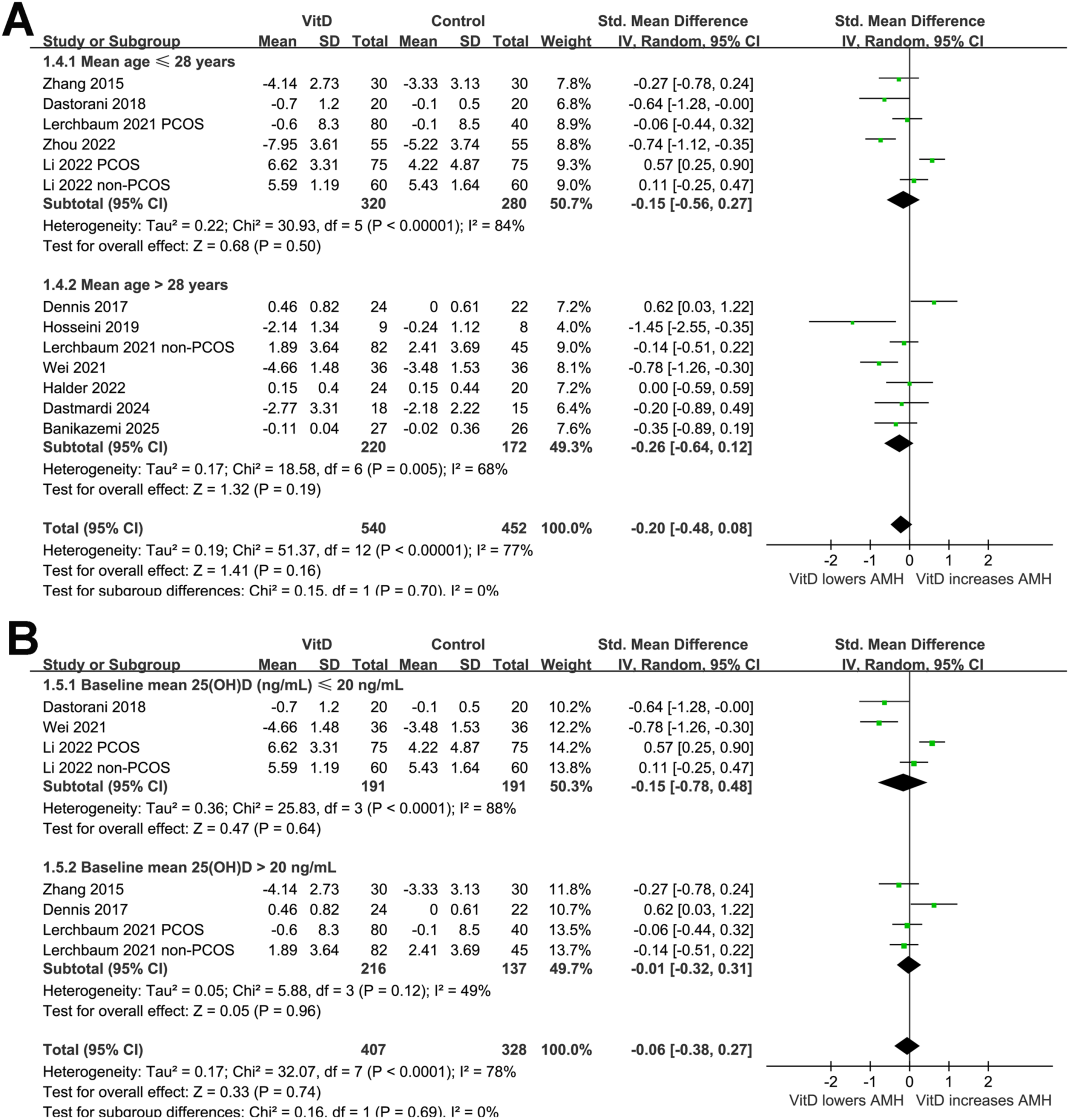
Forest plots for the subgroup analyses evaluating the influence of vitamin D supplementation on serum AMH level in women of reproductive age; **(A)** subgroup analysis according to the mean age of the women; and **(B)** subgroup analysis according to the baseline mean vitamin D level.

**Figure 4:**
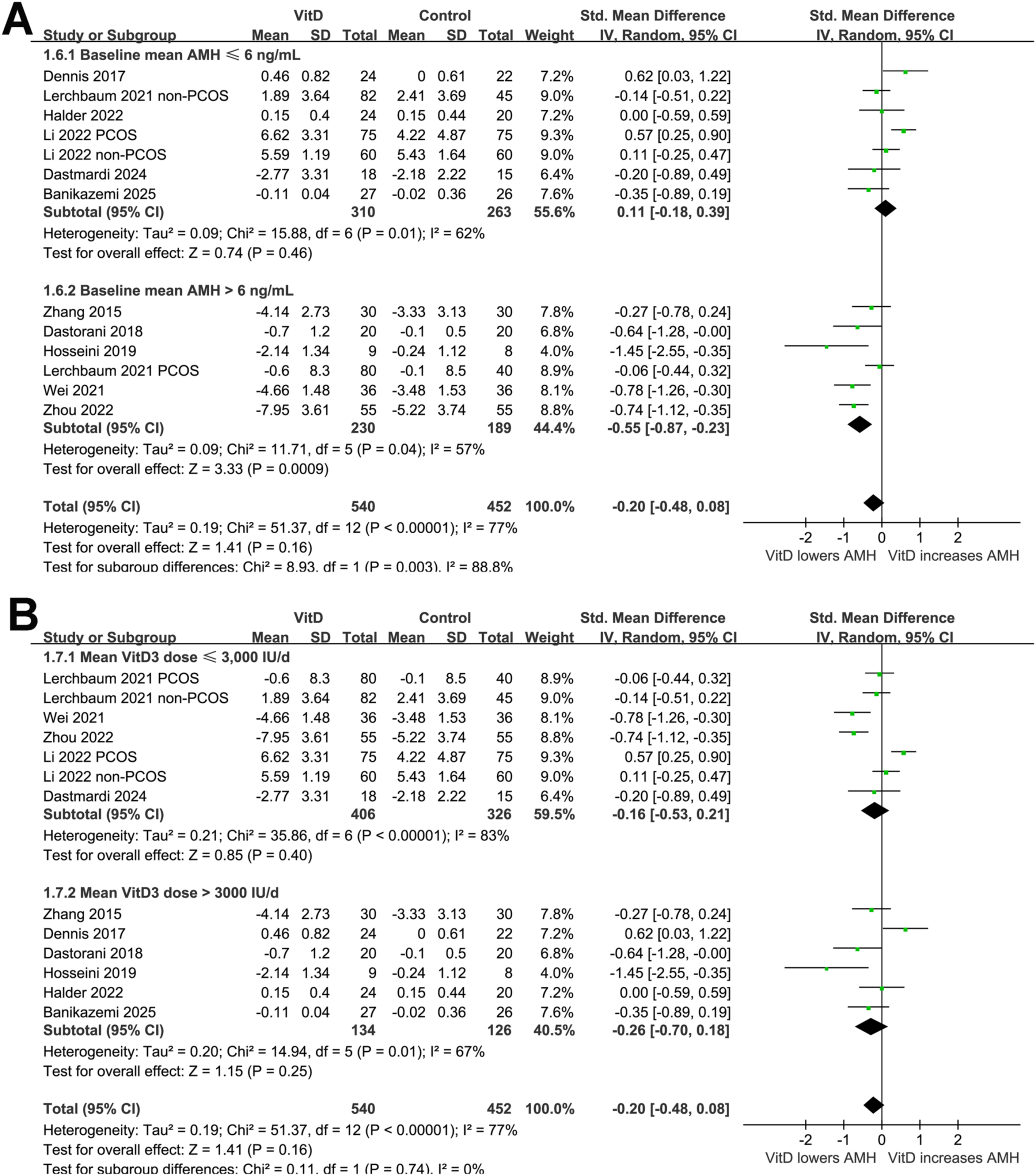
Forest plots for the subgroup analyses evaluating the influence of vitamin D supplementation on serum AMH level in women of reproductive age; **(A)** subgroup analysis according to baseline mean AMH level; and **(B)**, subgroup analysis according to the mean daily dose of vitamin D.

**Figure 5:**
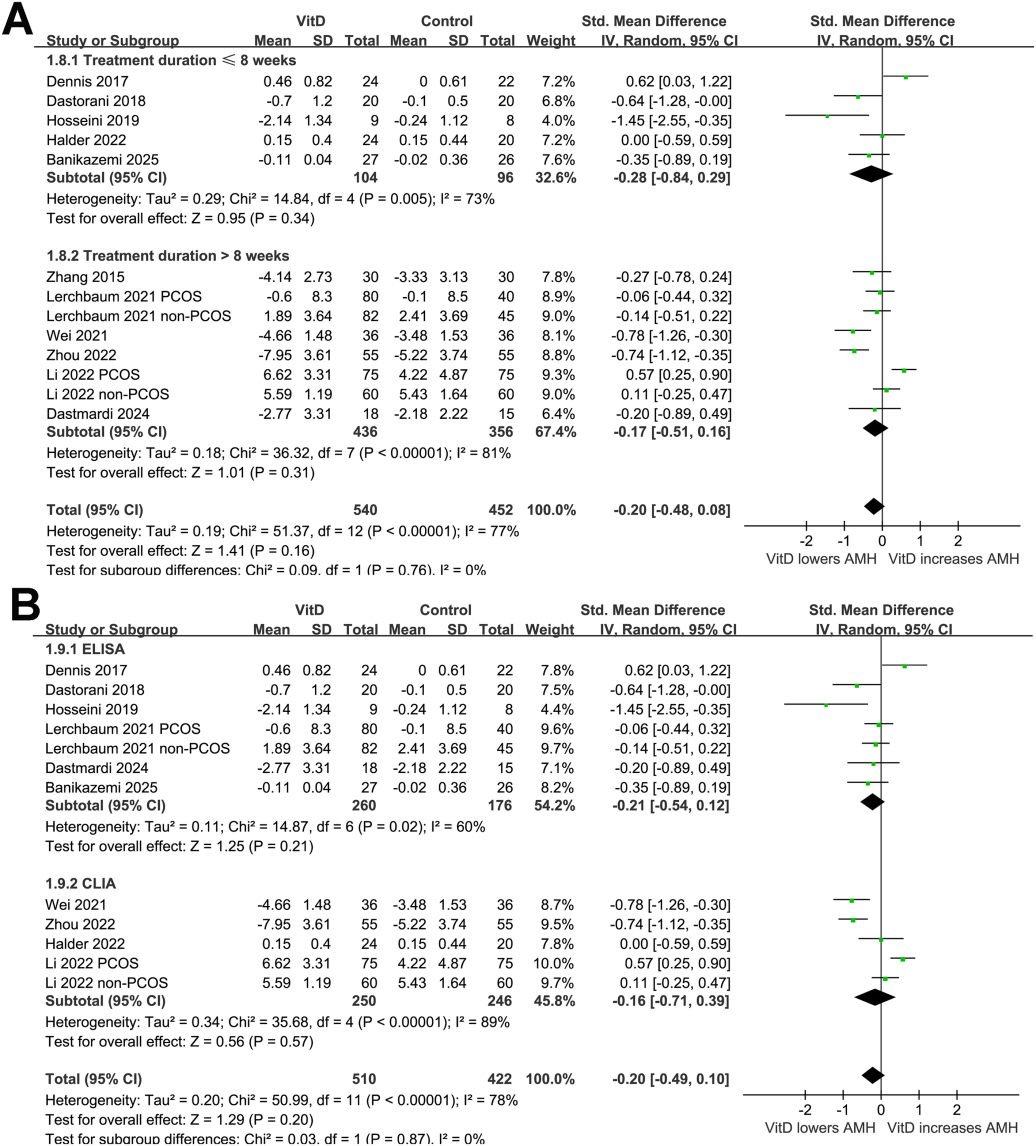
Forest plots for the subgroup analyses evaluating the influence of vitamin D supplementation on serum AMH level in women of reproductive age; **(A)**. subgroup analysis according to treatment duration; and **(B)** subgroup analysis according to the methods for measuring serum AMH.

In addition, further subgroup analysis suggested a significant interaction of study design on the effect of vitamin D supplementation on AMH level (*p* for subgroup difference = 0.02; **Figure 2B**). Vitamin D supplementation did not significantly affect the AMH level in double-blind trials (20–22, 27, 30), while was associated with a reduction in AMH in open-label trials (23–26, 28, 29). Further subgroup analysis suggested a trend of reduced AMH level after vitamin D supplementation in women with PCOS, but not in those without PCOS (SMD: -0.39 vs. 0.06, *p* for subgroup difference = 0.07; **Figure 2C**). The results did not seem to be significantly affected by mean ages of the women (*p* for subgroup difference = 0.70; **Figure 3A**), or baseline vitamin D level as indicated by 25(OH)D (*p* for subgroup difference = 0.69; **Figure 3B**). Interestingly, vitamin D supplementation significantly reduced the level of AMH in women with the mean baseline AMH > 6 ng/mL, but not in those with mean baseline AMH ≤ 6 ng/mL (SMD: -0.55 vs. 0.11, *p* for subgroup difference = 0.003; **Figure 4A**). Further subgroup analyses suggested that the results were not significantly affected by daily dose of vitamin D (*p* for subgroup difference = 0.74; **Figure 4B**), treatment durations (*p* for subgroup difference = 0.76; **Figure 5A**), or the methods for measuring the serum AMH (*p* for subgroup difference = 0.87; **Figure 5B**).

### 3.5 Publication bias

**Figure 6:**
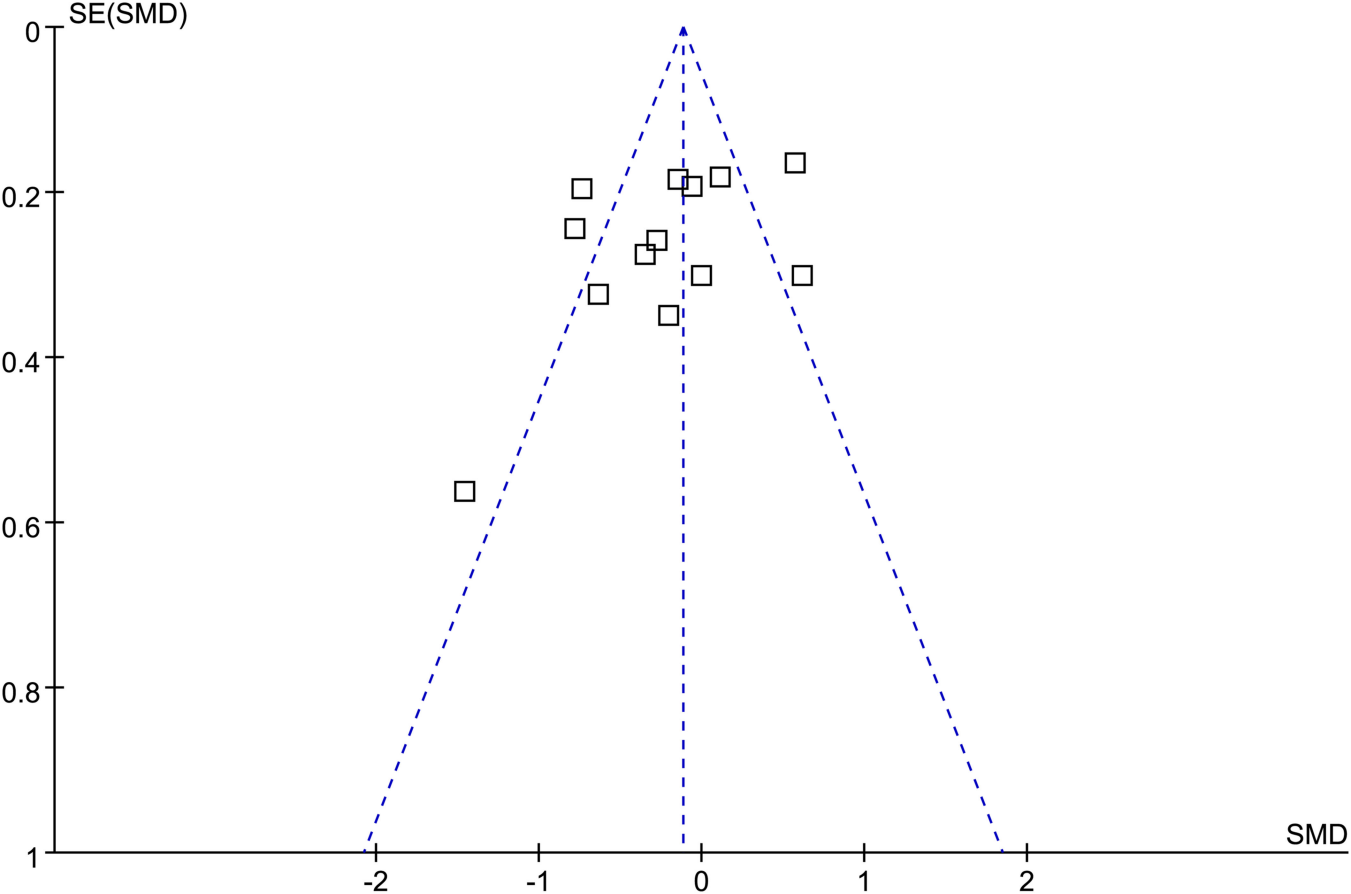
Funnel plots evaluating the publication bias underlying the meta-analysis of the influence of vitamin D supplementation on serum AMH level in women of reproductive age.

The funnel plots for the meta-analysis evaluating the effect of vitamin D supplementation on AMH are shown in **Figure 6**. Visual inspection suggested approximate symmetry, indicating a low likelihood of significant publication bias or small-study effects. Egger’s regression test did not detect statistically significant asymmetry (*p* = 0.54). However, given the limited number of included studies, these findings should be interpreted with caution.

### 3.6 Certainty of evidence

According to the GRADE assessment **Table 3**, the certainty of evidence for the effect of vitamin D supplementation on AMH was rated as moderate. Although all included studies were RCTs and no trial was judged as high risk of bias, the evidence was downgraded due to substantial heterogeneity (I² = 77%) and inconsistent effect directions across studies. Indirectness and imprecision were not considered serious concerns. Overall, vitamin D supplementation was unlikely to significantly affect AMH levels in women of reproductive age.

**Table 3:** Summary of findings and certainty of evidence (GRADE).

Outcome	No. of participants (studies)	Study design	Risk of bias	Inconsistency	Indirectness	Imprecision	Other considerations	Relative effect: SMD (95% CI),	Certainty of evidence (GRADE)	Comments
Influence of vitamin D supplementation on AMH	992 (11 RCTs, 13 datasets)	RCTs	Not serious – five trials were double-blind placebo controlled and six trials were open label. No trial was judged as of high risk.	Serious – direction of effect not consistent across studies; high heterogeneity (I^2^ = 77%) but not fully resolved in subgroup analyses.	Not serious – interventions, populations, and outcomes directly aligned with the review question.	Not serious – pooled CI not wide; total sample close to 1,000 provides adequate information size.	None.	-0.20 (-0.48 to 0.08)	⨁⨁⨁◯ Moderate	Vitamin D supplementation was not likely to significantly affect AMH.

GRADE, Grading of Recommendations Assessment, Development and Evaluation; RCTs, randomized controlled trials; AMH, anti-Müllerian hormone; SMD, standardized mean difference; CI, confidence interval;.

Specific reasons for each GRADE domain, including.

Risk of bias: Downgraded if a significant proportion of studies had unclear or high risk of bias in key domains (e.g., random sequence generation, allocation concealment, or selective reporting).

Inconsistency: Downgraded if substantial heterogeneity was observed (I² > 50%) and could not be explained by subgroup analyses or meta-regression.

Indirectness: Evaluated but not downgraded, as all included studies directly assessed the population and outcomes of interest.

Imprecision: Downgraded if confidence intervals were wide, or if the overall sample size was small.

Publication bias: Assessed using funnel plots and Egger’s test; downgraded if significant asymmetry suggested potential bias.

## 4 Discussion

This meta-analysis synthesizing evidence from RCTs indicates that vitamin D supplementation does not meaningfully improve serum AMH levels in women with DOR or related reproductive conditions. Although supplementation with vitamin D3 consistently increased circulating 25-hydroxyvitamin D concentrations in previous studies, this biochemical correction did not translate into a measurable enhancement of ovarian reserve as reflected by AMH. The overall direction and magnitude of effect were stable across sensitivity analyses, suggesting that the null finding is unlikely to be driven by a single influential study or methodological weakness. Collectively, the available randomized evidence does not support a clinically relevant role for vitamin D supplementation in restoring ovarian reserve as assessed by AMH.

Several biological considerations may explain why vitamin D supplementation failed to significantly increase AMH levels. Vitamin D receptors are expressed in ovarian granulosa cells, and experimental data suggest that vitamin D may modulate AMH gene transcription through vitamin D response elements in the AMH promoter region (36). In addition, vitamin D has been implicated in the regulation of folliculogenesis and steroidogenesis, and may influence granulosa cell function and sensitivity to follicle-stimulating hormone. However, AMH primarily reflects the quantity of small antral and pre-antral follicles, representing the growing follicle cohort (functional ovarian reserve), and does not directly quantify the primordial follicle pool (37, 38). In women with DOR, follicular depletion is often driven by age-related apoptosis, genetic susceptibility, autoimmune mechanisms, or prior ovarian injury (39). Such structural loss of follicular reserve is unlikely to be reversed by short-term hormonal or micronutrient interventions (40). Vitamin D may influence follicular function, steroidogenesis, or intra-ovarian signaling, but these effects may not be sufficient to increase follicle number or substantially alter AMH production (41). Moreover, vitamin D deficiency might represent a correlational biomarker rather than a causal determinant of diminished ovarian reserve (42). Observational associations between low vitamin D status and reduced AMH do not establish that correcting deficiency will restore ovarian reserve (41). This concept is reinforced by the most recent large-scale, hard-outcome RCT in women with PCOS undergoing IVF (43), which demonstrated that vitamin D supplementation (4000 IU/day for up to 90 days) significantly increased serum 25-hydroxyvitamin D levels but did not improve live birth rates. If vitamin D repletion does not enhance definitive reproductive outcomes such as live birth in a well-powered, multicenter trial, it is biologically plausible that it would also fail to meaningfully modify intermediate biomarkers such as AMH in women with compromised ovarian reserve. Therefore, changes in AMH may reflect alterations in follicular dynamics rather than true restoration of the underlying ovarian reserve.

The subgroup analyses provide additional insight. First, results stratified by study design (parallel versus other randomized structures) showed generally consistent findings, suggesting that methodological differences among trials are unlikely to explain the overall null effect. This strengthens confidence that the absence of benefit is not simply an artifact of trial design. Second, analyses according to PCOS versus non-PCOS populations are particularly informative. In women with PCOS, AMH levels are typically elevated due to increased follicle number and altered folliculogenesis (44). In this context, vitamin D might theoretically modulate AMH through effects on granulosa cell function or insulin resistance (45). However, even in PCOS subgroups, supplementation did not result in a consistent or clinically meaningful increase in AMH. In non-PCOS women, particularly those with true diminished ovarian reserve, AMH reflects reduced follicle quantity, and the lack of response to vitamin D is biologically coherent with irreversible follicular depletion (46). Third, stratification by baseline AMH levels did not identify a subgroup with clear benefit. Women with lower baseline AMH did not demonstrate a greater response to supplementation, which further argues against a restorative effect of vitamin D on ovarian reserve.

Several strengths of this meta-analysis merit emphasis. The literature search was comprehensive and up to date, and only RCTs were included, minimizing confounding and selection bias inherent in observational studies. The consistency of findings across sensitivity and subgroup analyses enhances the robustness of the conclusions. In addition, predefined subgroup analyses according to clinically relevant characteristics allow a more nuanced interpretation and reduce the risk of spurious *post hoc* inference. Nonetheless, important limitations should be acknowledged. Heterogeneity was present across trials in terms of participant characteristics, including age distribution, infertility etiology, and degree of ovarian reserve impairment. Interventions also varied in dose, duration, and baseline vitamin D status, which may influence responsiveness. The methods used to measure AMH differed among studies, including various immunoassay platforms, potentially contributing to between-study variability. Despite subgroup and sensitivity analyses identifying potential effect modifiers such as study design and baseline AMH levels, substantial residual heterogeneity remained, likely reflecting differences in clinical populations, intervention regimens, and measurement methods across studies. In addition, we included only studies published in English and Chinese, which may introduce potential language bias, although major Chinese databases were systematically searched to improve coverage. Because individual participant data were unavailable, we were unable to perform more refined analyses adjusting simultaneously for age, baseline vitamin D concentration, AMH assay method, and other confounders. Furthermore, AMH itself has limitations as a surrogate marker of ovarian reserve. Although widely used, AMH reflects follicle quantity rather than oocyte quality and does not directly predict live birth. Fluctuations in AMH may not necessarily translate into clinically meaningful reproductive improvements. Another important consideration is follow-up duration. Most included trials had relatively short supplementation periods and limited post-intervention follow-up. It is possible that longer-term correction of vitamin D deficiency, initiated earlier in the reproductive lifespan, might exert different effects. Baseline vitamin D status may also modify response, and women with severe deficiency could theoretically derive greater benefit. However, without individual-level data, such hypotheses remain speculative. Finally, as with any meta-analysis, the number of available trials remains modest, and small-study effects cannot be fully excluded.

From a clinical perspective, these findings suggest that routine vitamin D supplementation should not be recommended solely for the purpose of increasing AMH or reversing diminished ovarian reserve. Although AMH is widely used to reflect ovarian responsiveness, it remains a surrogate biomarker and does not directly predict fertility outcomes such as live birth or cumulative pregnancy rates. Therefore, the absence of a significant effect of vitamin D supplementation on AMH is unlikely to translate into meaningful improvements in reproductive success. Correction of vitamin D deficiency remains important for general health and may confer benefits for metabolic or endocrine function, but expectations regarding improvement in ovarian reserve or fertility potential should be tempered. Clinicians should counsel patients that while vitamin D supplementation is generally safe and appropriate when deficiency is present, it is unlikely to restore follicle quantity or substantially alter reproductive prognosis in women with established DOR. In clinical practice, management strategies should continue to focus on evidence-based reproductive interventions rather than relying on micronutrient supplementation to improve ovarian reserve. Future research should focus on well-designed, adequately powered randomized trials with standardized AMH assays, clearly defined DOR criteria, and longer follow-up durations. Studies incorporating individual participant data meta-analyses could better clarify effect modification by age, baseline AMH, and vitamin D deficiency severity. Importantly, future investigations should prioritize clinically meaningful outcomes such as live birth rather than solely intermediate biomarkers.

## 5 Conclusions

In conclusion, current randomized evidence indicates that vitamin D supplementation does not significantly increase AMH levels in women of reproductive age. Although biologically plausible mechanisms exist, they do not appear sufficient to overcome underlying follicular depletion. These findings should be interpreted cautiously in light of study heterogeneity and methodological limitations, but they do not support vitamin D supplementation as an effective strategy to improve ovarian reserve as measured by AMH.

## Data Availability

The original contributions presented in the study are included in the article/**Supplementary Material**. Further inquiries can be directed to the corresponding author.
