# Supplementary material for: Influence of vitamin D supplementation on ovarian reserve as reflected by anti-Müllerian hormone levels: a meta-analysis of randomized controlled trials
Source: Front Endocrinol (Lausanne). 2026 May 11;17:1832704. doi: 10.3389/fendo.2026.1832704 (PMC13199052; doi:10.3389/fendo.2026.1832704)
Supplement: Supplementary file 1 [file DataSheet1.docx]

Supplementary Material

# Supplementary Data

**Supplementary data 1.** **Detailed search strategy for each database**

**PubMed**

("Vitamin D"[Mesh] OR "Cholecalciferol"[Mesh] OR "Ergocalciferols"[Mesh] OR "Calcitriol"[Mesh] OR "Paricalcitol"[Mesh] OR vitamin D[Title/Abstract] OR vitamin D2[Title/Abstract] OR vitamin D3[Title/Abstract] OR cholecalciferol[Title/Abstract] OR ergocalciferol[Title/Abstract] OR alphacalcidol[Title/Abstract] OR alfacalcidol[Title/Abstract] OR calcitriol[Title/Abstract] OR paricalcitol[Title/Abstract] OR doxerocalciferol[Title/Abstract]) AND ("Anti-Mullerian Hormone"[Mesh] OR anti-Müllerian hormone[Title/Abstract] OR anti Mullerian hormone[Title/Abstract] OR AMH[Title/Abstract]) AND (randomized controlled trial[Publication Type] OR controlled clinical trial[Publication Type] OR randomized[Title/Abstract] OR randomised[Title/Abstract] OR RCT[Title/Abstract] OR randomly[Title/Abstract] OR placebo[Title/Abstract] OR allocation[Title/Abstract] OR allocated[Title/Abstract])

**Cochrane Library**

(MeSH descriptor: [Vitamin D] explode all trees OR MeSH descriptor: [Cholecalciferol] explode all trees OR MeSH descriptor: [Ergocalciferols] explode all trees OR MeSH descriptor: [Calcitriol] explode all trees OR MeSH descriptor: [Paricalcitol] explode all trees OR vitamin D:ti,ab,kw OR vitamin D2:ti,ab,kw OR vitamin D3:ti,ab,kw OR cholecalciferol:ti,ab,kw OR ergocalciferol:ti,ab,kw OR alphacalcidol:ti,ab,kw OR alfacalcidol:ti,ab,kw OR calcitriol:ti,ab,kw OR paricalcitol:ti,ab,kw OR doxerocalciferol:ti,ab,kw) AND (MeSH descriptor: [Anti-Mullerian Hormone] explode all trees OR anti-Müllerian hormone:ti,ab,kw OR anti Mullerian hormone:ti,ab,kw OR AMH:ti,ab,kw)

**Embase**

('vitamin d'/exp OR 'cholecalciferol'/exp OR 'ergocalciferol'/exp OR 'calcitriol'/exp OR 'paricalcitol'/exp OR vitamin d:ti,ab OR vitamin d2:ti,ab OR vitamin d3:ti,ab OR cholecalciferol:ti,ab OR ergocalciferol:ti,ab OR alphacalcidol:ti,ab OR alfacalcidol:ti,ab OR calcitriol:ti,ab OR paricalcitol:ti,ab OR doxerocalciferol:ti,ab) AND ('anti mullerian hormone'/exp OR 'anti müllerian hormone':ti,ab OR 'anti mullerian hormone':ti,ab OR AMH:ti,ab) AND ('randomized controlled trial'/exp OR random*:ti,ab OR placebo:ti,ab OR allocat*:ti,ab OR control*:ti,ab)

**Web of Science**

TS = (("vitamin D" OR "vitamin D2" OR "vitamin D3" OR cholecalciferol OR ergocalciferol OR alphacalcidol OR alfacalcidol OR calcitriol OR paricalcitol OR doxerocalciferol) AND ("anti-Müllerian hormone" OR "anti Mullerian hormone" OR AMH) AND ("randomized controlled trial" OR "randomised controlled trial" OR randomized OR randomised OR RCT OR randomly OR placebo OR allocation OR allocated OR control))

**Wanfang**

(维生素D OR 维生素D2 OR 维生素D3 OR 胆钙化醇 OR 麦角钙化醇 OR 阿法骨化醇 OR 骨化三醇 OR 帕立骨化醇) AND (抗缪勒管激素 OR 抗苗勒管激素 OR AMH) AND (随机 OR 随机对照 OR 随机分组 OR 安慰剂 OR 对照)

**China National Knowledge Infrastructure (CNKI)**

(维生素D OR 维生素D3 OR 胆钙化醇 OR 骨化三醇 OR 阿法骨化醇) AND (抗缪勒管激素 OR 抗苗勒管激素 OR AMH) AND (随机对照试验 OR 随机 OR 安慰剂对照 OR 随机分组 OR 对照研究)
